# Transgender and non-binary patient simulations can foster cultural sensitivity and knowledge among internal medicine residents: a pilot study

**DOI:** 10.1186/s41077-024-00284-5

**Published:** 2024-03-20

**Authors:** Charlie Borowicz, Laura Daniel, Regina D. Futcher, Donamarie N. Wilfong

**Affiliations:** 1 Center for Inclusion Health at Allegheny Health Network, Pittsburgh, PA USA; 2 The Simulation, Teaching, and Academic Research (STAR) Center at Allegheny Health Network, Pittsburgh, PA USA; 3 Social Work, University of Pittsburgh, Pittsburgh, PA USA

**Keywords:** Transgender, Non-binary, Simulation, Internal medicine, Resident education, Gender diversity

## Abstract

Transgender and nonbinary patients face unique healthcare challenges, such as harassment, discrimination, and/or prejudice, at higher rates than their cisgender counterparts. These experiences, or even the fear of these experiences, may push patients to delay or forego medical treatment, thus compounding any existing conditions. Such extraneous issues can be combatted through cultural sensitivity. The authors designed blended education consisting of an online module followed by a live simulation to educate and promote sensitivity. Internal medicine (IM) residents (*n* = 94) completed the module, which introduced them to transgender community terminology and medical disparities, and ways to incorporate affirming behaviors into their practice. Afterward, they engaged in a simulation with true transgender-simulated patients (SPs) — either trans-masculine, trans-feminine, or non-binary. Residents were expected to conduct a patient interview mirroring an intake appointment. Residents then engaged in a debriefing session with the lead investigator and the SP to reflect on the experience, receive feedback and constructive criticism, and ask questions. After the education, the residents’ knowledge significantly increased, *t*(66) = 3.69, *p* ≤ 0.00, *d* = 0.45, and their attitude toward members of the transgender community also increased significantly, *t*(62) = 7.57, *p* ≤ 0.00, *d* = 0.95. Furthermore, nearly all residents (99%) reported the training allowed them to practice relevant skills and was a worthy investment of their time. Nearly half (45%) of the residents who listed changes they will make to their practice pledged to ask patients for their preferred name and pronouns. Most comments were positive (75%), praising the education’s effectiveness, expressing gratitude, and reporting increased confidence. Results provided evidence that the education was effective in increasing IM residents’ knowledge and attitudes. Further research is needed to investigate the longitudinal effects of this education and to extend the education to a broader audience. The investigators plan to adapt and expand the research to other specialties such as gynecology and emergency medicine.

## Introduction

Transgender and gender diverse (TGD) patients face multiple barriers in accessing healthcare, many of which stem from systemic transphobia in society and a lack in trans competent provider training. TGD people report experiencing discrimination, harassment, and prejudice in healthcare settings causing many to stop seeking treatment until it is medically necessary [1, 2]. If providers or healthcare staff harbor negative beliefs or attitudes toward trans individuals, it is likely to result in a poor-quality health service. Encountering transphobia from care providers, such as judgmental statements relating to gender/health status, facial expressions of disgust, misgendering, or other harmful clinician behaviors, negatively impacts the care received and the TGD patient’s future health [3, 4]. Furthermore, the act of patients having to educate their provider about trans health during appointments is correlated with an increase in anxiety and depressive/suicidal thoughts [5]. Similar experiences have been reported across a myriad of healthcare settings including primary care, such as general internal medicine and gynecology, and specialty services like pediatrics and emergency medicine [4, 6, 7]. These interactions are undesirable and disincentivize TGD people from accessing care when they need it. Those who do remain engaged with the healthcare system and receive treatment often must take on the role of educator, informing their provider on their needs as a transgender patient. This likely occurs when medical students and providers do not have social exposure to TGD individuals and are not academically trained on their unique care needs [8]. The responsibility to inform medical professionals and caregivers about TGD inclusive healthcare should be shouldered by educational programs and not by the patients themselves.

Literature supports the notion that the incorporation of gender-related care and lesbian, gay, bisexual, trans and queer (LGBTQ +) health issues in medical educational content is correlated with an increase in provider comfortability in treating LGBTQ + patients and an enhanced awareness of gender and sexuality as it relates to clinical practice [9–11]. Informing providers on the differences between sex assigned at birth, gender identity, gender expression, and sexual orientation, is key in providing them the tools to properly communicate and treat TGD patients. When information is coupled with simulations that involve providers being placed in medical scenarios with a transgender patient, caregivers report improvement in knowing how to better discuss gender identity and health issues with trans patients [12, 13]. It is vital for medical professionals to know how to navigate topics or questions that can potentially be triggering for the patient, like discussing sexual history or asking about genitalia. This includes understanding how to ask questions related to transitions, their experiences as a trans person, and how to refer to certain body parts.

One study found that medical students felt that they were more capable of obtaining a patient’s preferred name, pronoun, and an appropriate patient history after participating in a simulation with a trans patient [14]. Implementing a LGBTQ + inclusive curriculum enabled students to identify other gaps in their training regarding gender and sexuality. Similar results were found in research that focused on nursing students. By participating in a simulation with trans patients, nursing students felt they became more aware of how to address health issues that trans people are at a higher risk for, such as “depression, generalized anxiety disorder, panic attacks, and risk of harm by others” [15]. Simulations featuring TGD-simulated patients have been proven to be an effective way of promoting inclusive care practices and fostering cultural competence in healthcare providers, especially nurses [16]. A limitation with many studies that investigate the efficacy of trans-inclusive simulations is that their post-simulation assessments are typically distributed within a few months of its conclusion. Future research should investigate the long-term effects of these programs and how frequently care providers need to take refresher modules or engage in additional simulations to remain competent in LGBTQ + issues. 

Researchers Baldwin et al. [17] were interested in learning how TGD individuals characterize “good” healthcare experiences by examining interactions between gender expansive patients and their healthcare providers. They found that healthcare experiences were categorized positively “when providers and staff used inclusive language, demonstrated their experience and education, and treated identity disclosure as routine” [17]. This information emphasizes the connections between provider knowledge and positive care outcomes for TGD patients. Acknowledging the identity of gender diverse individuals makes them feel more comfortable seeking medical care when necessary as they are less likely to incur discrimination.

There is a decidedly large gap in education that includes information on gender nonconforming individuals in LGBTQ + related curriculum and simulations. The transgender patients in these scenarios typically fit within the gender binary and identify as a male or female. Gender nonconforming (GNC) patients are at a higher risk of being denied gender-affirming care because they do not fit into the binary confines of what medical professionals consider to be “transgender” [18]. GNC patients have reported experiencing a higher rate of disrespect from providers than their binary trans counterparts [5]. It is important to recognize that not all GNC patients identify as transgender, but since their gender identity does not correspond with their sex assigned at birth, they may still want to engage in transition-related care. Moving forward, education, trainings, and simulations on gender-related care should include information on how to meet the needs of GNC people.

The present pilot study sought to improve these issues by providing education to first-year internal medicine residency students. The intervention was comprised of a training on topics relevant to the TGD community and gender-affirming healthcare, in addition to a simulation with a transgender patient. This study investigated how the implementation of a trans-inclusive training and simulation impacted the knowledge and attitudes of IM residents, with a focus on change in implicit bias toward TGD individuals. This preliminary study furthers simulation research as we utilize gender diverse patients and measure how provider knowledge and attitudes were impacted. Informed by previous evidence, we hypothesized that providing an educational module on trans topics in healthcare will improve provider knowledge and attitudes toward gender diverse patients.

## Materials and methods

The ethics committee overseeing this study is the Allegheny Singer Research Institute at Allegheny Health Network with the approval number of 2019–176. The IRB granted a waiver of need to document written informed consent. All authors have no competing financial interests to disclose.

Simulation development largely followed Kern’s six steps. These simulation scenarios were developed by first looking at national needs assessment of the TGD community and then at the information on lack of LGBTQ + training for medical education. With this information, the idea for adding a transgender health module to the current internal medicine curriculum was approved by the curriculum director. The goals were identified as follows:

1. Utilize proper terminology to describe the trans/nonbinary community.
2. Identify important components of care delivery for trans/nonbinary patients.
3. Demonstrate behaviors showing knowledge of trans/nonbinary patients in a simulated clinical setting.

Once the objectives were provided to the curriculum director, they were used to create simulated patient scenarios that would fulfill these goals. Scenarios were reviewed with the curriculum director, simulated patients, and employees of the simulation center to ensure feasibility and applicability. The researchers adapted several scales of trans inclusivity and added additional questions to include nonbinary people, since the original scales only included binary (male or female) trans people. The TGD community includes nonbinary people, so this was included as part of the simulation teaching. The researchers sought to determine whether simulation teaching influenced attitudinal responses to trans and nonbinary patient populations among internal medicine residents. The researchers created an evaluation tool that assessed knowledge, attitudes, and response to the simulation.

Internal medicine (IM) residents (*n* = 94) watched a 40-min pre-recorded video on transgender community terminology and medical disparities and ways to incorporate affirming behavior into their practice. Residents completed this online module on their own electronic devices, at their convenience prior to their participation in the simulation. Residents were not required to complete the surveys.

Next, the residents engaged in a live simulation with genuine transgender-simulated patients (SPs) who were actively recruited from the local urban community in which the research took place. SPs were selected through social media recruitment via one author’s social networks. Prior simulations had requested the use of transgender SPs, so many of the cohort were pulled from that existing pool. That pool came from the social network of one of the researchers and used word of mouth from those individuals to recruit others. A social media post was shared in multiple LGBTQ + spaces.

Each simulation was held in a mock exam room in the simulation center and featured one of three patient demographic types: trans-masculine, trans-feminine, or non-binary. Residents were randomly assigned to a simulation, with equal placement probabilities. Although the gender identity of each SP differed, all scenarios followed the same protocol with identical objectives. The two objectives were to increase IM residents’ knowledge of transgender terminologies and disparities and to stimulate and/or nurture cultural sensitivity and inclusion.

All scenarios utilized a patient name on the medical chart that differed from the patient’s chosen or affirmed name and gender identity/pronouns that were incongruent with the person’s legal sex on the chart. Each SP had completed multiple steps toward gender affirmation and was currently participating in one potentially high-risk behavior (e.g., unprotected sex with multiple partners). Residents were expected to elicit these patient details through good rapport building skills housed in a harm reduction framework, which is particularly important for marginalized and/or vulnerable patients. Other information included on the medical chart was the patient’s chief complaint/reason for visit and date of birth. Residents were instructed to conduct a thorough patient interview that mirrored an intake appointment for primary care, without a physical examination. The SPs were instructed to behave and respond as they normally would in this type of encounter. For example, the SPs were instructed to only disclose information if/when they felt comfortable. The simulation was video recorded, and the lead investigator observed the simulation through a one-way glass. After the simulation, residents then engaged in a debriefing session with the lead investigator and the SP to reflect on the experience, correct any misinterpretations, receive performance feedback, and explore their emotions, thought processes, and actions. Debriefing sessions were not recorded, and data was not collected. While not documented with any rigor, many of the feedback and debriefing conversations followed a similar format. For each simulation, the SP’s comfort was of utmost importance. One common theme was whether the SP felt they were asked appropriate questions at the appropriate time, such as their gender identity and pronouns. Some residents chose to ask those questions when discussing the reason for the visit, which may or may not have been related to gender. For example, one patient coming in with complaints of fatigue and muscle aches explained that while they appreciated the questions about gender identity for respect purposes, the questions were not asked at the appropriate time. Residents were also given feedback on their use of language, specifically whether residents had used the language used by patients, including correct name and pronouns but also extending to body parts.

Another theme was whether the resident felt they had enough information to give the patient. This varied widely as the cohort spanned 3 clinical years. In future iterations, the individual clinical years will have simulations more tailored to their degree of experience, incrementally increasing in complexity. Psychological safety was a main consideration during these encounters. Debriefing began with a question to the resident (“How did you feel during that interaction?”) and then built out from there. This was an opportunity for residents to indicate whether they felt uncomfortable during the simulation, and why. The facilitator and SP led the following discussion with feedback that focused on the positive elements of the interaction and then offered recommendations for improvement. This was framed in a way of capitalizing on strengths and also encouraging the existing skills. For example, in a simulation where the resident had excellent bedside manner but limited clinical knowledge, the conversation would go similar to this one below (paraphrased from actual encounters):

Resident: I just don’t know about dosing hormones and how to manage that, it made me feel awkward.

Facilitator: The clinical knowledge will come with time and experience, and it’s okay to admit that you need to do more research. What I liked about your approach is that you were comfortable explaining that to the patient, and assured them you would get the information and follow up with them.

SP: I felt comfortable that you would get accurate information, instead of referring me to another provider when I already made an appointment with you.

These approaches were designed to support the resident’s psychological safety. Because this is a growing and evolving field, that ambiguity can cause psychological discomfort in residents. When residents would make an error in the discussion, it would be addressed in a nonjudgmental, clinically focused way. Another paraphrased example is below:

Facilitator: I recall you saying that you would plan to monitor hormone levels every month. The clinical guidelines recommend monitoring every 3 months for the first year, and annually once hormone levels are stable.

The facilitator and SPs made efforts to include only objective feedback in terms of behavior, statements, and clinical guidelines. SPs were discouraged from using subjective statements in terms of their interpretation of the resident’s behavior or attitude, although they were allowed to explain how they felt as a result of the encounter.

Knowledge and attitudinal data were collected confidentially before and after the residents participated in the education. Participants’ responses were electronically recorded with the final four digits of their phone numbers to decrease acquiescence and social desirability bias. Electronic anonymity was aimed to promote honest responses. Responses were recorded in Qualtrics, an online experience management platform.

Knowledge regarding transgender identities and healthcare was assessed through a 20-item pre/posttest which was comprised of 15 multiple-choice items and 5 true/false items. Knowledge scores could range from 0 to 20, with higher scores indicating more knowledge. This scale was reviewed by two master’s level content experts and a PhD-level psychometrician for validity evidence by examining word choice and item structure for potential complexities, biases, and/or ambiguities. The scale’s reliability was evaluated through the test/retest coefficient, *r* = 0.53, *p* ≤ 0.00, which was statistically significant. Changes in knowledge before and after the simulation were analyzed with a paired sample *t*-test.

Attitudes were assessed through an internally created scale of 20 items to which the residents marked their agreement levels on 5-point Likert scales. The scale was adapted from Billard [19] but altered to more accurately represent nonbinary identities and gender diversity. All items were written in the positive direction wherein more agreement meant a more positive attitude toward the transgender community. All items were worded positively to reduce cognitive load [20]. Total attitude scores could range from 20 to 100. This scale was reviewed by a master’s level content expert and a PhD-level psychometrician for validity evidence by examining word choice and item structure for potential complexities, biases, and/or ambiguities. The scale’s reliability was evaluated through Cronbach’s alpha which was 0.91, which can be interpreted as very high. Changes in attitude before and after the simulation were analyzed with a paired sample *t*-test.

Participants’ reaction to the education was measured with a blended evaluative tool of 10 items. This survey reflected two levels of Kirkpatrick’s new world model [21], reaction and learning. Seven items were closed and asked residents to mark their agreement levels to positively worded items on 5-point Likert scales. Three items were open and asked residents to record the most beneficial aspect of the training, what changes they will make to their practice, and other comments. Evaluative data were also recorded anonymously and confidentially through the Qualtrics platform. This evaluation survey was reviewed by two master’s level content experts and a PhD-level psychometrician for validity evidence by examining word choice and item structure for potential complexities, biases, and/or ambiguities. The evaluation data was analyzed using frequency and proportional descriptive statistics.

## Results

Among residents who completed both the knowledge pretest and posttest (*n* = 67), over half of them (55%) earned higher scores on the posttest after the simulation education. A paired sample *t*-test was conducted to determine the effect of the simulation education on knowledge scores. The results indicated there was a significant difference between knowledge scores before the training (*M* = 17.64, *SD* = 1.25) and knowledge scores after the training ((*M* = 18.22, *SD* = 1.39), *t*(66) = 3.69, *p* ≤ 0.00), with a moderate effect size, *d* = 0.45. The two items with the largest increases in percent correct before and after education rose by more than 6%. The percentage of residents who correctly answered a terminology item regarding a method of gender dysphoria alleviation increased by 7%, and the percentage of residents who correctly answered an item regarding feminizing hormones increased by 8%.

Among residents who completed both the before and after attitudinal scale (*n* = 63), most residents (84%) exhibited higher, more positive attitude scores after the simulation education. A paired sample *t*-test was conducted to determine the effect of the simulation education on attitudinal scores. The results indicated there was a significant difference between attitudinal scores before the training (*M* = 87.44, *SD* = 10.33) and attitudinal scores after the training ((*M* = 92.30, *SD* = 7.94), *t*(62) = 7.57, *p* ≤ 0.00), with a large effect size *d* = 0.95. Item statistics were examined to determine which items experienced the largest gain in positive attitude scores. Table 1 presents the three items with means that increased by at least 0.4 before and after the education.

**Table 1:** Attitudinal items with the largest increases in item statistics

Item	Mean		Median	
	Before	After	Before	After
I believe there are many genders, not only male and female	3.75	4.25	4	5
Transgender people seem completely normal to me	4.16	4.64	4	5
Transgender people are natural	4.14	4.55	4	5

Nearly, all residents submitted a course evaluation (*n* = 92). Virtually, all respondents (*n* = 91, 99%) reported the training was an opportunity to practice relevant skills and was a worthy investment of their time. After the education, most residents (*n* = 88, 96%) reported they were confident in providing transgender patients with compassionate, competent care, and most respondents (*n* = 86, 93%) believed that this training helped them see transgender people in a more positive light.

When the residents were asked what changes they would make to their practice after taking this course, residents pledged to continue to educate themselves, to state their own pronouns first, and to ensure the patient pronouns are properly documented in the medical records. However, the most reported change among the responding residents (45%) was to ask for the patient’s preferred name and pronouns. The open-ended comments on the evaluations were mostly positive (*n* = 37, 75%), followed by nearly a quarter of suggestions (*n* = 11, 22%), and a single negative comment (*n* = 1, 2%).

The positive comments included praise for the effectiveness of the training, gratitude for the experience, and feelings of increased confidence. Most of the suggestions written on the evaluations (*n* = 7, 64%) requested more training sessions. One resident (9%) suggested changing the course format to multiple sessions, “This might be better set up in a multiple occurrence to allow for a visit to address a real case as well as a time dedicated to discussing a treatment plan.” Two residents (18%) suggested clearer directions for the simulation, and one resident (9%) asked to blind simulation participants from knowing that the patient is transgender. The single negative comment expressed technical difficulties with the pre-course video.

## Discussion

IM residents benefitted from receiving education in the form of an online learning module and simulation consecutively. Residents increased their knowledge about transgender communities and showed more positive attitudes toward transgender people after the training. Many practicing providers cite a lack of education or knowledge as a barrier to care for trans patients [22]. This training sought to alleviate this barrier and encourage providers to broaden their scope of practice to include gender diversity. Current findings suggest that exposure to and interaction with trans patients in conjunction with a self-directed learning module improve knowledge and attitudes of residents toward trans people.

During debriefing, most SPs were asked if they would return to this doctor for follow-up if it was a real appointment. This led to a discussion of whether rapport was built between the resident and the SP. Because most simulations included recommendations for follow-up appointments, it was helpful to discuss whether the SP would feel comfortable returning for additional care. A quarter of trans people (25%) avoid care because of mistreatment or fear of mistreatment (James et al., 2016), so this question was used to reflect upon what could have gone differently in the event of a “no” or “maybe” answer. Data was not collected on the distribution of SP answers but used only to facilitate discussion.

Researchers attempted to eliminate bias by creating de-identified, matched sets for the tests. Scoring was done by entering the correct answers into Qualtrics and reporting out. There were no subjectively graded elements in the assessment.

A similar study may inform future research to add more academic rigor. Weingartner et al. [23] conducted simulations in which the patient’s gender identity was the only changing factor. In contrast, the current study changed the circumstances of the patient’s appointment/chief complaint with each changing SP. The Weingartner study was able to compare each scenario based on objective criteria since the chief complaint, behavior, requests, and follow-up recommendations were consistent across SPs. Furthermore, the SPs in this scenario were equally likely to be cisgender or transgender, therefore testing the residents’ likelihood to ask for pronouns and affirmed names, and connect the need for health screening related to assigned sex at birth. While the current study had many of these elements present in individual simulations, this was not standardized or analyzed. In a future iteration of this study, these encounters being standardized with only one variable changing would allow for comparative analysis of treatment based on gender identity (whether nonbinary patients are treated differently than binary patients, whether trans people are provided with the same recommendations as their cisgender counterparts, etc.). Another study conducted by Noonan et al. [24] did not inform the learners that they were participating in a transgender health module but used the same approach of changing only gender identity between SPs. This approach allows for comparison of treatment for cisgender and transgender patients, with the reason for the visit and recommendations remaining the same. This was not feasible for the current study since the residents were assigned to a transgender health module that included a simulation. The learners were aware they were entering into a simulation about trans and gender diverse patients. We prepared learners by giving them a dead name (legal name) that differed from the patient’s preferred (affirmed, chosen) name, as well as an age, sex, and chief complaint. Despite having prior knowledge that they would be working with a transgender patient, not all learners incorporated affirmed name and/or pronouns without prompting. Part of the lesson for these learners is that any patient may have a preferred name, and that should be asked of anyone regardless of gender identity. Debriefing also included recommendations for asking preferred name, pronouns, and identity, if this was not addressed during the simulation. Many of the scenarios involved some steps of transition, to highlight the differences among the trans community; for example, one of the simulated patients was using hormones bought online, another patient had a history of gender-affirming surgery, while others had none of these things.

To prepare simulated patients, the educator provided pre-briefing in which the scenario was discussed and any emotional triggers addressed. All of the patient actors were transgender or gender diverse and had prior negative healthcare experiences due to their gender identity. The facilitator explained that the actor may choose to end the scenario at any time if they became upset or felt unsafe. Actors were told that they should respond the same way as they would in an actual healthcare visit. This may include getting emotional, shutting down, withholding information, becoming standoffish, or responding with education and patience. All of these responses depended on the learner’s approach, rapport building, and patient’s comfort level. Actors in these scenarios were able to use their lived experience to provide feedback to the learners. Simulated patients in these types of scenarios should always be people of lived experience, in this case, people who are transgender or gender diverse. This allows the actors to respond authentically and provide effective feedback.

The educator and facilitator of this scenario were also a transgender person, who was able to provide additional third-party feedback from observation of the scenario, also based on prior lived experience. This gave the learner two perspectives and highlighted the diversity of experience within a marginalized group. For educators who are not transgender or gender diverse, these researchers recommend using objective criteria and allowing the simulated patient to relay the information related to being a gender diverse person; for example, a third-party observer can indicate when and if a learner asked the simulated patient for their name and pronouns, whether they provided all the relevant information for follow-up, or make observations about their body language. Educators and facilitators should work with TGD people whenever possible to develop and implement scenarios that reflect lived experiences. Local LGBTQ + organizations often include educators that will work with healthcare organizations and medical schools. Scenarios should be reviewed by TGD persons who are compensated for their time.

It should be noted that in future studies where the SP pool is limited, every effort should be made to include TGD individuals in the SP pool. The increase in virtual provider appointments creates an environment in which virtual simulated appointments can directly mimic a new patient appointment, albeit without the physical exam components. If the local SP pool does not include TGD individuals, researchers are encouraged to reach out to local LGBTQ + organizations for guidance and recruitment and consult with national and international educators as needed. It is not recommended that non-LGBTQ + SPs are used in place of people with lived experience. The exposure to, and interaction with, TGD individuals is critical to learning and allows for true feedback from a historically marginalized individual. It is the opinion of these authors that people external to these marginalized groups would not be able to respond authentically to a situation they have not experienced.

An advantage of simulation for all SPs but especially for those from marginalized communities is the opportunity to reverse the power dynamics of a medical encounter and provide education to the learner, a medical professional. A grounded theory of stigma by Poteat et al. [25] discusses the impact of, and contributing factors to, power dynamics during a healthcare interaction. This theory postulates that providers enter into interactions with transgender patients with ambivalence and uncertainty due to the lack of medical education on this topic. This shifts the expertise, and by extension the power, to the patient. The discomfort leads providers to try to assert their authority by engaging in discriminatory behavior or reinforcing stigma. Resistance of interpersonal stigma by providers, and internalized stigma by patients, can challenge those existing power dynamics. In the current study, debriefing supported this model by showing how learners with less experience working with transgender patients tended to misgender and/or use the dead name of the patient, which is considered mistreatment. Resident learners were asked what they think went well during the encounter and what they feel they could have improved. This allowed them to offer suggestions for improvement first, and then the SP offered constructive feedback. Many learners were reminded that it is acceptable to not have every answer in a field of medicine that is still developing, therefore reducing the need to engage in stigmatizing behavior to reinforce the power dynamics. More effectively managing the uncertainty contributes to provider comfort and resists the need to engage in discriminatory behavior to assert authority. Much of the debriefing was spent discussing the patient relationship and how that could be preserved and/or improved.

What we understand as a result of this study is that TGD-simulated patients are able to provide learners with a unique experience and critical information about treating someone of a gender minority group. We also learned that being exposed to and interacting with TGD-simulated patients increase positive feelings toward this marginalized group. During debriefing, many participants shared that they were grateful to have these conversations because of “being afraid of offending” their patients. They welcomed the opportunity to ask questions in a low-stakes environment. Many negative attitudes toward trans patients were a by-product of lack of knowledge; once that knowledge increased, so did the more positive attitudes. The exposure to, and opportunity to interact with, TGD people is an important component of challenging bias. This also shows that these attitudes are malleable and not steadfast; often when we think about bias and discriminatory behavior, the assumption is that those are unchanging. This research shows that even with a small intervention, attitudes toward marginalized groups can change significantly. Further research should explore the degree to which behavior changes after a change in knowledge and/or attitudes, as well as whether these changes remain over time.

Future research is also needed to determine if these approaches would be effective with practicing providers who are not in their residency, as well as other medical staff such as nurses, medical assistants, and surgical techs. Future research should also include other residencies such as obstetrics and gynecology, emergency medicine, family medicine, and neurology. Further exploration of intersecting identities could be helpful in determining whether simulated patient interactions are effective in reducing bias based on race, disability, and other identities in addition to gender.

### Limitations

This study was conducted on a single site for a single residency group of varying levels of experience (years 1–3 of internal medicine residency). The variance in experience was not considered. There was also no longitudinal evaluation to determine whether results were salient over time and whether changes in attitude were enduring. The measures used were developed internally and lacked the benefit of repetition and validity through multiple uses.

Potential confounding factors in the research include the fact that residents knew they were preparing for a transgender health simulation, the simulation was observed and discussed beforehand (without details of the clinical case), and that some residents had interacted with trans people to varying degrees in their clinical rotations. The pool of residents included 3 years of residency, so the experience was highly variable among participants. For example, a resident in their first year would have much less clinical acumen than a resident in their third year. Stratifying interventions either based on the year of residency or self-reported experience working with trans patients could help eliminate this confounding variable. To eliminate the confounding factor associated with residents being made aware of the subject matter of the simulation, this would need to be one of a series of encounters with both cisgender and transgender patients presenting for varying medical concerns. This also reduces or eliminates the ability to measure whether our SPs were more or less likely to be denied healthcare based on binary vs. nonbinary gender identity. The residents understood that they were to treat the SP like an actual patient and under observation were unlikely to turn away an individual for healthcare. It is the belief of the researchers that these conditions cannot be replicated in a research setting. Further field study is needed to understand the reasons for denial based on gender identity and offer strategies for improvement.

Researchers also did not evaluate the effect of SP responses on resident attitudes. While SPs were given general guidelines to begin the interaction in a neutral fashion, they were also instructed to respond as if this was a real encounter. None of the SPs became aggressive or hostile toward the resident, although some became withdrawn if the questions were too invasive or the SP did not feel the line of questioning was conducted with interpersonal sensitivity. Further research could include qualitative interviews with residents that asked about the elements that produced a shift in attitudes, if applicable.

Researchers did not record whether SPs stated their desire to return to the provider for two reasons: this was not considered objective data but rather a point of discussion to guide the debriefing, and to use this meaningfully, the researchers would have had to match these responses to survey results, eliminating the anonymity of survey results. In future versions of this study, it would be beneficial to include qualitative SP interviews that are separate from the debriefing and link those through the same identifiers used for the pre/post surveys.

## Conclusion

In conclusion, this study demonstrated that providing a trans-inclusive healthcare education module to IM residents improved knowledge about and attitudes toward the transgender population. Next steps in furthering this research include following up with providers who went through the training to see how they incorporated the information into their practice. Assessing for longitudinal effects is crucial as many studies in this area, including our own, are short term. Were the positive attitudes toward TGD patients long lasting? Did they feel more comfortable/confident treating trans patients? These are just a few questions that will reveal the effectiveness of how TGD inclusive simulations translate to real-life encounters with patients. This work could also be extended to other relevant specialties (obstetrics and gynecology, emergency medicine, pediatrics) and professions (emergency medical responders, nurse practitioners, physician assistants). Additionally, we hope to engage in collaborative research with other health systems, cities, or programs to further promote healthcare inclusive of all gender identities. This simulation model can serve as a foundational basis for other healthcare networks to expand upon ensuring both trans and gender diverse patients receive quality care. Providing trans-inclusive continuing education for IM residents and to new residents each year will improve provider knowledge and attitudes toward the TGD community. Doing so will increase the likelihood of TGD patients having affirming primary care experiences. Outpatient practices will likely see an increase in patient satisfaction and positive healthcare outcomes as TGD individuals will feel more comfortable seeking medical care when necessary.

## Data Availability

The datasets during and/or analyzed during the current study are available from the corresponding author on reasonable request.
